# Myosin Head Configurations in Resting and Contracting Murine Skeletal Muscle

**DOI:** 10.3390/ijms19092643

**Published:** 2018-09-06

**Authors:** Weikang Ma, Henry Gong, Thomas Irving

**Affiliations:** BioCAT, Department of Biological Sciences, Illinois Institute of Technology, Chicago, IL 60616, USA; wma6@iit.edu (W.M.); hgong7@hawk.iit.edu (H.G.)

**Keywords:** skeletal muscle, X-ray diffraction, actomyosin interaction, sarcomere structure, super-relaxed state

## Abstract

Transgenic mouse models have been important tools for studying the relationship of genotype to phenotype for human diseases, including those of skeletal muscle. We show that mouse skeletal muscle can produce high quality X-ray diffraction patterns establishing the mouse intact skeletal muscle X-ray preparation as a potentially powerful tool to test structural hypotheses in health and disease. A notable feature of the mouse model system is the presence of residual myosin layer line intensities in contracting mouse muscle patterns. This provides an additional tool, along with the I_1,1_/I_1,0_ intensity ratio, for estimating the proportions of active versus relaxed myosin heads under a given set of conditions that can be used to characterize a given physiological condition or mutant muscle type. We also show that analysis of the myosin layer line intensity distribution, including derivation of the myosin head radius, R_m_, may be used to study the role of the super-relaxed state in myosin regulation. When the myosin inhibitor blebbistatin is used to inhibit force production, there is a shift towards a highly quasi-helically ordered configuration that is distinct from the normal resting state, indicating there are more than one helically ordered configuration for resting crossbridges.

## 1. Introduction

Synchrotron small-angle X-ray diffraction is the method of choice for nm-scale structural studies of actively contracting muscle from living and skinned muscle preparations in concert with mechanical data, such as muscle force and length changes, in real physiological time. This approach has told us much of what is known of the temporal sequence of events in muscle activation [1,2,3,4,5], crossbridge behavior during defined muscle states [6,7,8], insect muscle during stretch activation [9,10], and the molecular mechanism of myofilament length-dependent activation [11,12,13]. More recently, however, there has been a shift in focus towards attempting to understand the molecular basis of muscle disease states, and how this can inform novel therapeutic strategies for these often-times untreatable diseases. This new direction for the field has led to a burgeoning of functional and biochemical work using mouse models of inherited myopathies but, so far, with little emphasis on understanding how disease mutations in sarcomeric proteins alter their structural dynamics leading to the functional phenotype (see, for example, [14]).

There are very few reports [15,16], however, of X-ray diffraction of murine skeletal muscle in the literature to indicate how useful a preparation this can be for biophysical studies of the relationship of mutations of sarcomeric proteins to disease phenotype. One such phenotype is the maximum activated force per unit area, often deficient in disease. One physiological parameter that can be used to explain deficiencies in force production is the fraction of myosin heads that are attached to actin, and available to generate force. X-ray diffraction has the potential to provide such information but, hitherto, these inferences have been indirect, with considerable variability between estimates arrived at by different means [17,18,19].

The dispositions of myosin heads under resting conditions will influence how they will behave in subsequent contractions. Under resting conditions, the myosin heads have been proposed to be in either the so-called “super-relaxed” (SRX) state, or the disordered-relaxed state (DRX). The SRX state has been characterized as a state where myosin has a much lower ATPase rate than under normal conditions [20], explaining the low energy consumption in resting muscle. Structurally, myosin heads in the SRX state have been proposed to adopt the so-called interacting-head motif (IHM) where one of the two heads of a myosin molecule is folded back on to its own coiled-coil S2 tail [21,22,23]. In contrast, in the DRX state, myosin heads are presumed to be disordered and freely moving in the myofilament lattice with ATPase activity as high as with isolated myosin [24]. Due to being sequestered by the IHM, myosin heads in the SRX state are less likely to be recruited when muscle contracts, compared to myosin heads in the DRX state [25,26].

Blebbistatin and BTS (*N*-benzyl-p-toluene sulfonamide) are small molecules that inhibit force production in myosin II [27,28] and have been used for many years in structural and biochemical studies. In particular, blebbistatin has been shown to stabilizes the SRX state in skeletal muscle [21] as well as cardiac muscle [29] while BTS does not show such an effect. Treatment with blebbistatin appears to stabilize the IHM, most obviously in isolated thick filaments [30,31], but also in fibers [29]. Blebbistatin, therefore, can be a useful tool to investigate the SRX state in muscle and the role of IHM in muscle regulation. It remains to be established, however, whether myosin heads in the SRX state necessarily adopt the IHM. A fundamental question that remains to be answered is whether the SRX state is the only ordered myosin relaxed state in skeletal muscle, or whether there are intermediate quasi-helically ordered states between the SRX and DRX states.

Our experiments examining mouse skeletal muscle were motivated, in part, by previous experiments done in collaboration with Dr. Massimo Reconditi and his colleagues at the University of Florence, to examine the effects of temperature on the X-ray patterns on mammalian skeletal muscle. These results will appear elsewhere. Here, we show that intact whole murine EDL (extensor digitorum longus muscle) and soleus muscles can yield high quality 2D X-ray fiber diffraction patterns, very similar to those obtained from the more widely studied frog leg muscles. As a consequence, almost any scientific question one could address with frog skeletal muscle, can now be addressed with murine skeletal muscle, but with the advantage of being able to access the wide range of transgenic mouse models that either have been created in the past or could be, in the future, used to address the structural basis of disease phenotypes. In our diffraction patterns from mouse EDL and soleus muscles, the myosin layer lines characteristic of relaxed muscle, while less intense, were still visible in the contracting pattern, and the intensity of the layer lines was inversely related to the active tension generated. We were also able to show that, unlike intact frog muscle [17,32,33], but like skinned rabbit psoas muscle [34] and intact cardiac muscle [35], the change in the ratio of the intensity of the 1,1 and 1,0 equatorial reflections, (I_1,1_/I_1,0_), showed a near-linear relationship to tension, especially during early phases of tension generation. By comparing how the intensity of the 4th myosin layer line (MLL4) changes between rest and contracting at different values of active tension, we were able to characterize the relationship between the MLL4 intensity change and the number of myosin heads that were recruited during contraction. The residual MLL4 intensity during isometric tetanic contraction suggests that less than half of the myosin heads in mouse skeletal muscle are recruited from the resting configuration during normal tetanic contraction under our conditions. The near-linear relationship between the equatorial intensity ratio change and active tension suggests that the majority of the recruited myosin heads were attached to actin. Using blebbistatin to stabilize myosin heads in the SRX state, we found that, besides the highly ordered SRX state, there is at least one other ordered relaxed myosin state in mouse skeletal muscle that can give rise to myosin-based layer lines in X-ray fiber diffraction patterns.

## 2. Results

### 2.1. X-ray Patterns from Mouse EDL Muscle

**Figure 1:**
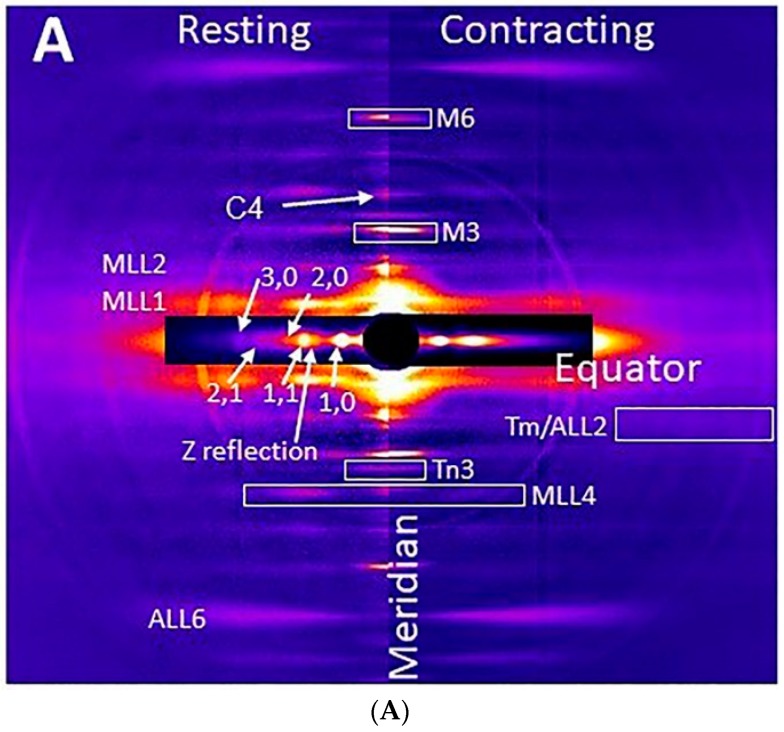

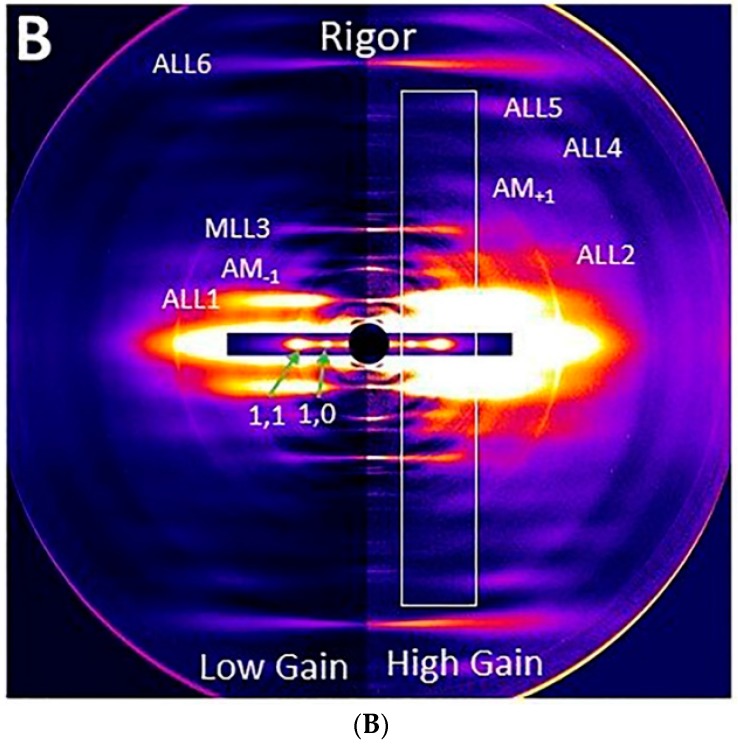
X-ray diffraction patterns from mouse EDL muscle. (**A**) Patterns from resting (left) and contracting (right) mouse EDL muscle. The equatorial reflections and myosin layer lines (MLL) are as indicated. (**B**) X-ray pattern from muscle in rigor at low gain (left) and at high gain (increased 3-fold, right). The equatorial reflections and actin-based layer lines (ALL) are as indicated. The box indicates the range used for the integrated intensity trace shown in Figure 2. C4: 4th myosin binding protein C reflection; M3: third order myosin meridional X-ray reflection; M6: sixth order myosin meridional reflection. AM: actomyosin.

The 2D X-ray diffraction patterns from resting mouse muscle (Figure 1) are generally similar to those obtained from the much better studied intact frog leg muscles or skinned rabbit psoas muscle. Diffraction patterns from resting muscles show prominent layer lines (indicated by MLL1 and MLL2 in Figure 1A) based on the 43 nm myosin thick filament repeat. While some lattice sampling can be seen in lower angle MLLs, MLL4 (indicated in Figure 1A) appears to be unsampled as it is in skinned rabbit psoas muscle [36]. Also indicated in Figure 1A are the myosin-based meridional reflections M3 (14.3 nm), whose intensity arises primarily from myosin heads, and M6 (7.2 nm), whose intensity arises primarily from the thick filament backbone [2,37,38]. Both these reflections move inwards towards the center of the pattern when muscle contracts, consistent with observations from previous studies in frog muscles [1,2]. Although not visible in Figure 1, interference splitting can be seen in the M3 reflection in X-ray patterns taken with a longer camera and a higher resolution CCD detector (data not shown) as previously reported for frog muscle [38]. The equatorial reflections are well-resolved, extending beyond the 3,0 equatorial reflection in the resting pattern (Figure 1A, left). The actin helix consists of 13 G-actin subunits in 6 turns of the helix with a pitch of 35.9 nm [39]. This gives rise to a series of actin layer lines (ALL) with the 5.9 and 5.1 nm being most prominent. These layer lines can be observed in both resting and contracting muscle patterns. In contracting muscle patterns, the outer part of ALL2, which we will call the Tm/ALL2 reflection, was visible at 19.3 nm^−1^ (Figure 1A, right). MLLs are no longer visible and the ALLs, weak in resting muscle, are strong and located at a higher axial spacing in the rigor pattern (Figure 1B).

The radial distribution of intensity in ALL6 shifts inwards towards the meridian in the rigor pattern due to the binding of myosin to actin as expected from previous studies on frog [40] and rabbit muscle [7].

The muscle length where muscle generated maximum tension (*Lo*) was 10.59 ± 0.1773 mm, *n* = 37 for the EDL muscles and 9.758 ± 0.1303 mm, *n* = 18 for the soleus muscles. The sarcomere lengths at *Lo* were 2.591 ± 0.03839 µm, *n* = 29 for EDL and 2.781 ± 0.05009 µm for soleus, *n* = 28. This difference is consistent with the shorter thin filaments EDL (1.04 µm) than in soleus (1.14 µm) [41].

**Figure 2:**
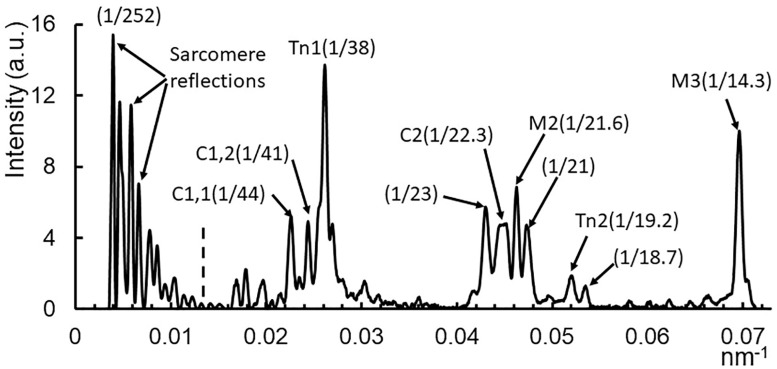
Low order meridional reflections in resting muscle. The meridional reflections, including high order sarcomere repeat, M1 cluster, M2 cluster, and M3 reflection. Taken from resting muscle with a 9 m sample to detector distance. Integration region is from 0.03 nm^−1^ to 0.077 nm^−1^ in reciprocal space (white box in Figure 1B). Reflection intensities to the left of the dotted line are scaled down by a factor of 15 for visibility. C1: lowest angle myosin binding protein C reflection; C2: second myosin binding protein C reflection (doublet with C1); M2: second order myosin meridional X-ray reflection; M3: third order myosin meridional X-ray reflection; Tn1: 1st order troponin reflection; Tn2: 2nd order troponin reflection.

The low order meridional pattern from resting muscle is shown in more detail in Figure 2. In this image, taken with a 9 m camera, one can see a family of myosin-based meridional reflections based on the 43 nm myosin repeat. In addition to the aforementioned M3 reflection, there are clusters of reflections near the position of the “forbidden” M1 and M2 myosin reflections at 43 and 21.5 nm, respectively. These reflection clusters include the Tn1, Tn2, and Tn3 (Figure 1) reflections representing the 1st, 2nd, and 3rd order reflections respectively of the troponin axial repeat of 38.7 nm along the thin filament. The clusters also contain reflections arising from myosin binding protein C. These appear as a series of doublets, presumed to be the result of interference between the two half-sarcomeres, with each pair (C1, C2, and C4 are visible in Figure 2) indexing on an ∼44 nm repeat. At very low angles, one can see the ~10th to 20th orders of the sarcomere repeat. The sarcomere reflection intensities are scaled down by a factor of 15 in order to be visible in the figure.

**Figure 3:**
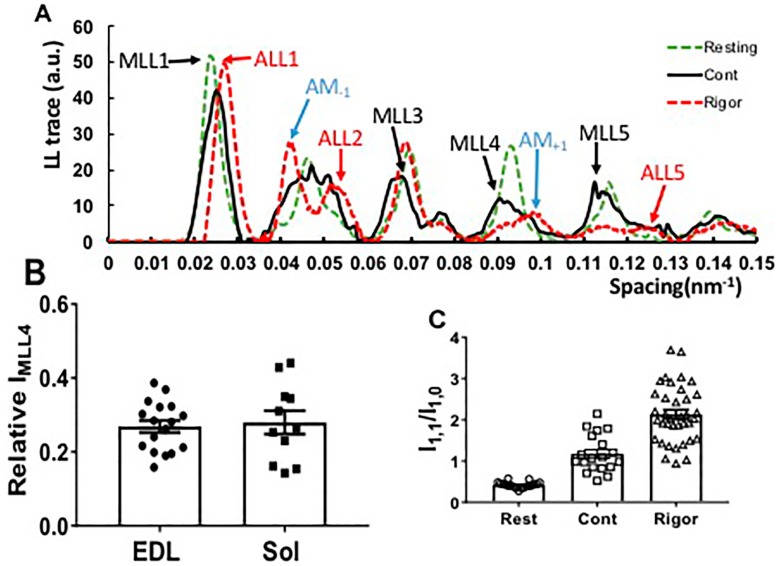
Residual myosin layer line (MLL) intensity in X-ray patterns from contracting mouse muscle. (**A**) The layer line intensity traces from resting (green arrows), contracting (black arrows) and rigor patterns (red arrows) along the meridian integrated over a radial spacing range from 0.03 nm^−1^ to 0.077 nm^−1^. Both myosin (MLL) and actin (ALL) layer lines were present in contracting patterns, while all myosin layer lines were replaced by actin- or actomyosin-based layer lines in rigor patterns. (**B**) MLL4 remained at about 30% of its resting value in both EDL (0.27 ± 0.02, *n* = 17) and soleus muscle (0.28 ± 0.1, *n* = 11) during the plateau region of tetanic contraction. (**C**) Equatorial intensity ratio in resting (Rest), contracting (Cont) and rigor (Rigor) muscle.

To examine the layer lines more closely, a radially integrated intensity trace (from 0.03 nm^−1^ to 0.077 nm^−1^ in reciprocal space, white box in Figure 1B) parallel to the meridian was generated from the resting, contracting, and rigor EDL muscle X-ray patterns as shown in Figure 3A. In the rigor patterns, there was no visible myosin-based layer lines in EDL muscle (Figure 1B), being replaced by ALL and AM layer lines at slightly larger axial spacings (Figure 3A). The intensity of MLL1 was much stronger than the rest of the layer lines, and was scaled down by a factor of 2 in resting pattern and 4 in rigor pattern, in order to be visible in the figure.

In rigor patterns (Figure 3A), the ALL traces (red dashed line) were at a longer axial spacing compared to resting MLL traces (green dashed line) from resting patterns. Two AM reflections at ∼1/24 nm^−1^ (AM_−1_) and at ∼1/10.3 nm^−1^ (AM_+1_) were seen in rigor patterns. These observations are consistent with previous studies on frog sartorius muscle as well as rabbit psoas muscle [42,43]. In the contracting patterns, the apparent MLL1 (black dashed line) was actually a mixture of MLL1 and ALL1, which intensifies during contraction [40] so that it appears to shift slightly towards higher spacing. In addition to the myosin meridionals, the higher order MLLs, namely MLL4, MLL5, and MLL6, in contracting patterns move inwards, consistent with the ~1% increase in myosin axial spacing in the thick filaments seen in mouse muscle [44] which can be compared to the ~1.5% increase seen in frog muscle [45,46] during contraction.

### 2.2. Residual Myosin Layer Line Intensity in Contracting Muscle Patterns

Unlike frog muscle, in which all MLLs were replaced by a different set of ALLs and actomyosin-based lines (AM), when muscle contracts [43], the myosin layer lines characteristic of the resting state were still visible in fully activated, tetanically contracting mouse muscle (Figure 1A). Figure S1 shows a two-dimensional X-ray pattern of M4–MLL4–AM_+1_ area. In contracting muscle, the MLL4 is much stronger and is azimuthally offset from the AM_+1_ actomyosin layer line (0.097 nm^−1^ for AM_+1_ and 0.093 nm^−1^ for MLL4). The two layer lines are also centered at different radial spacings. MLL4 was centered at a radial spacing of 0.062 nm^−1^, while AM_+1_ was centered at 0.075 nm^−1^. AM_+1_ is much weaker than the residual resting MLL4 in maximally contracting patterns. In the contracting trace shown in Figure 3A, a small bump is visible in the MLL4 profile at the AM_+1_ position, which is seen more clearly in the rigor trace suggesting that although there will be a contribution from the AM_+1_ intensity to the MLL4 measurements, it will be small under normal contracting conditions.

The remaining MLL4 intensity during maximal contraction was about 30% of the resting state intensity in both EDL and soleus muscle (0.27 ± 0.02, *n* = 17, in EDL, and 0.28 ± 0.03, *n* = 11 in soleus (Figure 3B). This residual layer line intensity in contracting patterns indicates that a substantial fraction of the myosin heads remained in a quasi-helically ordered resting configuration during the fully activated state. To support this notion, and to validate that the reflections in contracting patterns were actually residual resting myosin layer lines, we also examined the rigor state where all myosin heads are presumed to be bound to actin [19]. The equatorial intensity ratio (I_1,1_/I_1,0_), an indicator of the proximity of myosin heads to actin, was 0.421 ± 0.01 (*n* = 19) in resting EDL muscle, 1.175 ± 0.1 (*n* = 19) in contracting EDL muscle, and 2.13 ± 0.11 (*n* = 37) in rigor EDL muscle (Figure 3C). The large difference in I_1,1_/I_1,0_ between contracting and rigor is consistent with a substantial fraction of the myosin heads remaining in the resting configuration rather than in close proximity to actin during tetanic contraction.

### 2.3. Effect of Myosin Inhibitors on Myosin Layer Lines

**Figure 4:**
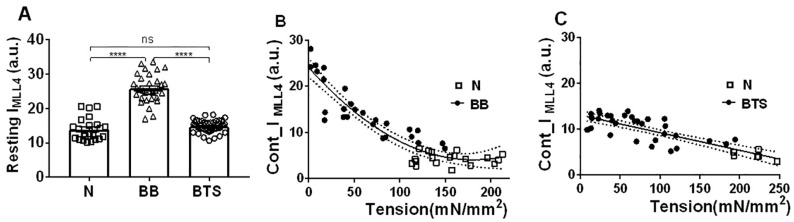
Myosin layer line 4 (MLL4) intensities (in arbitrary units–a.u.) in resting and contracting mouse EDL muscle. (**A**). The normalized MLL4 intensities were the same in untreated normal (N) muscle and BTS-treated muscle but the intensities almost doubled in samples after blebbistatin treatment (BB). ns: *p* ≥ 0.05; ****: *p* < 0.0001. (**B**) MLL4 intensities against tension generated by blebbistatin-treated (BB) and normal untreated (N) muscle. Line is the fit to a second order polynomial with *R*^2^ = 0.87. Dotted lines show 95% confidence limits (**C**) MLL4 intensities against tension generated by BTS-treated and normal muscle. Solid line is the linear fit with *R*^2^ = 0.68 and the dotted lines are 95% confidence limits. The slope of the line is significantly different from zero (*p* < 0.0001).

To quantify the relationship between residual MLL intensity and a wide range of active tensions, blebbistatin and BTS, two small molecule myosin inhibitors, were used to vary the levels of activation in mouse EDL muscle. In the experiments reported in Figure 4B,C, the muscles were incubated in 75 µM blebbistatin and 100 µM BTS, respectively, for varying periods of time, to achieve varying levels of tension. Blebbistatin has been shown to enhance the resting myosin layer line intensities in skinned rabbit fibers [47]. The same result was seen in intact mouse EDL muscle. The normalized MLL4 intensities in resting muscle almost doubled with blebbistatin treatment (25.81 ± 0.68; *n* = 30) (Figure 4A). The MLL4 intensities in muscle treated with BTS (14.74 ± 0.27 nm; *n* = 40) were not significantly different from resting, untreated (normal) muscle (13.93 ± 0.66; *n* = 24). The MLL4 intensities were inversely correlated to the tension generated by the muscle contraction in patterns from muscle treated with both blebbistatin (Figure 4B) and BTS (Figure 4C). Incubation with blebbistatin or BTS did not significantly change the width of the MML4 in the axial direction. The MLL4 intensity decreases in a non-linear manner (fit to a second order polynomial with *R*^2^ = 0.87) with an increase in active tension in blebbistatin-treated muscle, while this relationship is linear (*R*^2^ = 0.69) in BTS-treated muscle with a slope of −0.03839 ± 0.00432 that is significantly different from zero (*p* < 0.001). It should be noted that the parabolic function was chosen in Figure 4B merely to emphasize the non-linearity of the relationship. It has no other physical significance.

The distribution of maximum isometric tension at the beginning of the experiment, *To*, varied widely from muscle to muscle. This can be seen graphically in Figure 4B,C as the distribution of tensions for untreated muscle (N). When these results were averaged, *To* was 170.4 ± 7.892 mN/mm^2^, *n* = 26.

### 2.4. Equatorial Intensity Ratios

**Figure 5:**
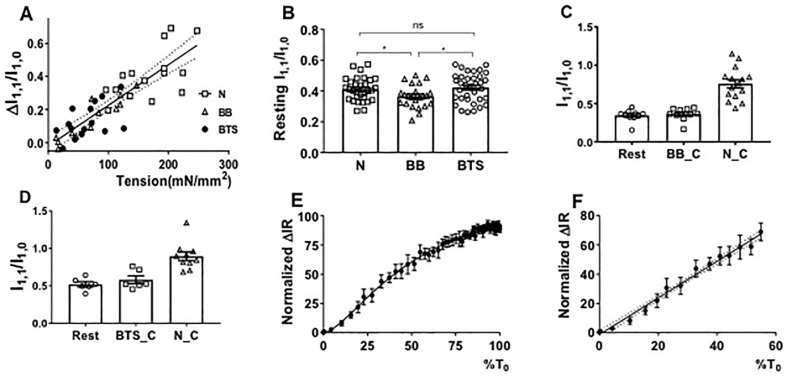
Equatorial intensities in active and resting mouse EDL muscle. (**A**) The change in I_1,1_/I_1,0_ (ΔI_1,1_/I_1,0_), has a linear relationship with the tension generated by blebbistatin-treated (BB), BTS-treated (BTS), and normal contracting muscle (N). (**B**) I_1,1_/I_1,0_ in resting normal muscle (from Figure 3B), blebbistatin-treated (BB), and BTS-treated muscle. ns: *p* ≥ 0.05, *: *p* < 0.05. (**C**) Intensity ratio changes from resting to contracting conditions at maximum inhibited (less than 10% of normal contraction tensions) by BB as compared to normal contracting (N_C). (**D**) Intensity ratio changes from resting to contracting conditions at maximum inhibited (less than 10% of normal contraction tensions) by BTS as compared to normal contracting (N_C). (**E**) ΔI_1,1_/I_1,0_ (ΔIR) as a percent of its maximal value, as a function of normalized tension during tension rise after stimulation in tetanically contracting muscle in the absence of inhibitors. (**F**) As C, but showing linear behavior at low tension values.

The reduction of force generated by the muscle treated by blebbistatin and BTS was proportional to the treatment time, indicating that it took some time to diffuse and penetrate into the whole intact muscle. The equatorial ratio change, ΔI_1,1_/I_1,0_, (I_1,1_/I_1,0_ in contracting minus I_1,1_/I_1,0_ in resting muscle), when muscle contracts in blebbistatin-treated, BTS-treated, and untreated muscle, showed a linear relationship with active tension (*R*^2^ > 0.75, slope = 0.0025 ± 0.00018) (Figure 5A). As observed previously in resting muscle [48], the I_1,1_/I_1,0_ values were smaller in blebbistatin-treated muscle compared to values from BTS-treated and untreated normal muscle (Figure 5B), indicating that myosin heads were closer to the thick filament backbone in the presence of blebbistatin. There was no significant increase in I_1,1_/I_1,0_ when the muscle was activated in a fully inhibited state (less than 10% of normal tetanic tension) with either blebbistatin (Figure 5C) or BTS (Figure 5D). Figure 5E shows ΔI_1,1_/I_1,0_, from untreated contracting muscle as a function of active tension normalized to its maximum value. This curve deviates from linearity at high tension with a Hill model providing a significantly better fit (*p* < 0.001, *n* = 6) than did a linear fit. However, at low tensions, up to 60 percent of the maximum tension generated by the muscle, ΔI_1,1_/I_1,0_ shows a linear relationship (slope = 1.254 ± 0.03821) to normalized tension, with *R*^2^ equal to 0.99 (Figure 5F).

As has been observed previously [49], the radial widths of the equatorial reflections peaks are wider in contracting muscle than in resting muscle (Table 1). Both σ_s_ and σ_d_ (see methods) are approximately twice as large in contracting muscle than in resting and this difference is statistically significant. The axial widths of the reflections appear to be ~7% larger in contracting muscle, but this difference was not significant in paired *t*-tests.

### 2.5. Effect of Myosin Inhibitors on Radial Spacing of Myosin Heads

**Figure 6:**
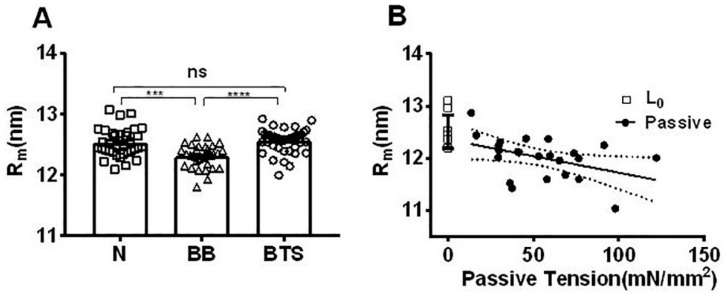
Average myosin head radius (R_m_) in resting mouse EDL muscle. (**A**) Under resting conditions, R_m_ was the same in normal muscle (N) and BTS-treated muscle, but smaller in samples with blebbistatin treatment (BB). ns: *p* ≥ 0.05, ***: *p* < 0.001 and ****: *p* < 0.0001. (**B**) At optimal length (L_0_) in passive stretching experiments, the R_m_ was 12.5 ± 0.29 nm (*n* = 11) and decreases with increasing passive tension. Solid line is the linear fit with *R*^2^ = 0.68 and the dotted lines are 95% confidence limits. The slope was significantly different from zero (*p* < 0.05).

The radial position of the center of mass with respect to the center of the thick filament can be estimated from the position of the first maxima on the myosin layer lines MLL1 and MLL4 [12,50,51]. The average myosin head radius (R_m_), so estimated (Figure 6A), was smaller with blebbistatin treatment (12.31 ± 0.04 nm, *n* = 30) when compared to R_m_ in normal muscle (12.53 ± 0.04, *n* = 35), indicating that blebbistatin brought myosin heads closer to the thick filament backbone. This is consistent with the equatorial intensity ratio data (Figure 5B). R_m_ estimated from the residual layer lines in contracting untreated muscle (12.58 ± 0.11 nm, *n* = 19) was unchanged from the resting value (*p* = 0.58). The average myosin head radius from BTS-treated muscle, however, was the same as normal muscle in both resting (12.56 ± 0.03 nm, *n* = 40) and contracting states (12.47 ± 0.04 nm, *n* = 36) (Figure 6A).

### 2.6. Effect of Passive Tension on Radial Head Position

To observe the effect of passive stretch on radial head position, the muscle was initially set to L_0_ and R_m_ (12.5 ± 0.28 nm, *n* = 11) was the same as seen in resting muscle as described above (Figure 6A). Figure 6B shows that R_m_ decreases linearly with increasing of passive tension with a slope of −0.006 nm–mm^2^/mN (*p* < 0.05) indicating that myosin heads move closer to the backbone at longer sarcomere length as has been observed previously in intact cardiac muscle [12].

### 2.7. Effect of Blebbistatin on Tropomyosin Movement

**Figure 7:**
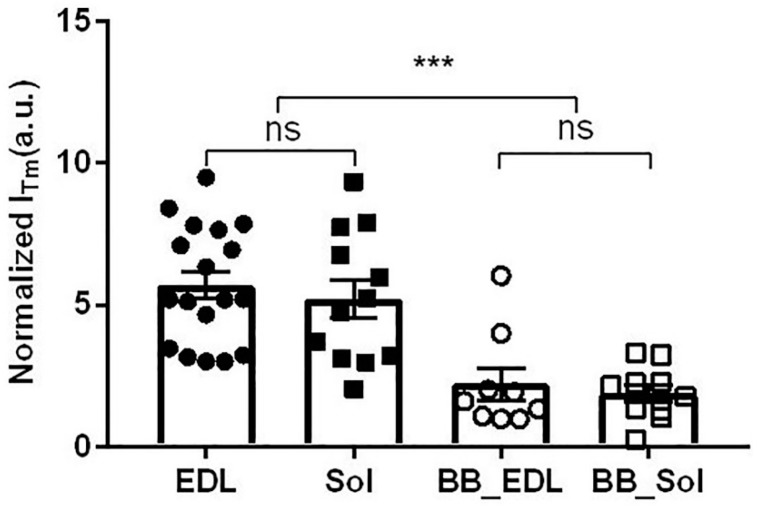
Normalized integrated intensities from Tm reflections from both normal contracting and fully blebbistatin inhibited muscle. EDL and Sol—untreated EDL, and soleus muscles respectively. BB_EDL and BB_Sol—maximally inhibited (<10% maximum force) EDL and soleus muscle, respectively. ***: *p* < 0.001.

The outer part of the second actin layer line is known to intensify during contraction [52,53,54] due to the movement of tropomyosin on the surface of the actin filament unblocking the myosin binding sites on actin. Figure 7 shows that there is no significant difference in integrated intensity of the outer part of the second actin layer line (Tm/ALL2) between normal contracting soleus (5.2 ± 0.7; *n* = 12) and EDL (5.7 ± 0.5; *n* = 18) muscle. The integrated intensities of the Tm/ALL2 were, however, significantly smaller in fully blebbistatin inhibited muscle (2.2 ± 0.6; *n* = 9 (EDL) and 1.9 ± 0.3; *n* = 10 (soleus) than in normal contracting muscle.

## 3. Discussion

### 3.1. Residual Relaxed Myosin Layer Lines Present during Isometric Contraction

When frog muscle contracts, the myosin-based layer lines completely disappear, being replaced by a new set of actomyosin-based layer lines [43,55]. This has been interpreted as most of the myosin heads leaving the ordered resting state to be available to interact with actin during tetanic contraction [17,32]. While diffraction patterns from resting and contracting mouse muscle were otherwise very similar to frog muscle patterns, in contracting mouse EDL or soleus muscle, the myosin-based layer lines were still visible at fully activated state, as shown in Figure 1. Myosin layer lines are observable in previously published X-ray patterns from contracting mammalian muscle [14,16,56,57] but have not previously been commented on or analyzed. MLL4 is well resolved from actin layer lines, and lacks substructure indicative of lattice sampling that would complicate analysis, as previously observed in skinned rabbit psoas muscle above 20 °C [36], and was, therefore, chosen to study crossbridge behavior under contracting conditions. The radial spacing of the first maxima on MLL4 was the same in resting and contracting muscle, confirming that the myosin-based layer line only came from quasi-helically ordered myosin heads in the resting configuration.

Under normal tetanic contraction, about 28% of the resting MLL4 intensity remained in both EDL and soleus muscle. Based on the assumption that X-ray diffraction intensity is proportional to mass squared, this indicates that more than half (~53%) of the myosin heads remain in the resting configuration during tetanic contraction. This assumption may turn out to be an oversimplification since myosin heads are not point diffractors, and the shape of the heads are changing when binding to actin, but this calculation allows an indication of the likely trend. While the force is adjusted by incubation with blebbistatin for various time periods, the relationship is non-linear, and the relationship between force and MLL4 intensity is linear when BTS is used instead. We discuss the likely reasons for this discrepancy below.

There may be concerns that the residual MLL4 intensities in contracting muscle are due to incomplete activation or that they are misidentified actin (ALL) or actomyosin (AM) reflections arising from the interaction of myosin to actin [43]. Since the experiments were performed at room temperature (22 °C), the X-ray patterns were sharp and clear as expected at 20 °C and above [36]. The stimulation voltages and currents were optimized for every preparation to ensure maximum activation and the contracting tetanic tensions were comparable with previous experiment done at 37 °C from mice of roughly the same age and size [58]. Furthermore, published X-ray diffraction patterns (see Figure 4 of [16]) from skinned mouse EDL single fibers showed similar residual myosin layer lines at pCa4, where maximum isometric contraction occurs. It seems highly unlikely, therefore, that the residual MLL4 intensity seen in contracting muscle patterns is due to incomplete activation.

Figure 3A shows that the residual MLL4 in contracting muscle is well separated from any actin layer lines that will have a different axial spacing. To eliminate the possibility that the putative residual MLL4 was an actomyosin (AM) reflection arising from myosin bound to actin [43], we compared the layer line axial traces from resting, isometrically contracting and rigor muscle in Figure 3A. The intensity profiles showed a clear difference in MLL spacing in resting/contracting patterns (Figure 3A green and black dashed lines) and ALL spacing in rigor patterns (Figure 3A red dashed lines). The lower order layer lines (first and second) in contracting pattern were a mixture of MLL and ALL. The higher orders MLL (MLL4 and MLL5) moved towards to lower axial spacing in contracting patterns, compared to rested pattern, while retaining the same radial intensity profile. This inward axial shift is expected from the ~1% axial spacing increase in the activated myosin thick filament [44,59]. This indicates that the residual MLL layer lines arise from active thick filaments, not incompletely activated ones. Therefore, it does not seem that the residual MLL4s have been misidentified.

### 3.2. Estimation of Fraction of Actin-Bound Myosin Heads during Contraction

In the cross-section, the myofilaments in the A-band form a 2D hexagonal crystal lattice with thick filaments at the lattice positions and thin filaments at the trigonal points. In this lattice, the so-called 1,0 crystallographic lattice planes contain only thick filaments, and the so-called 1,1 planes contain both thick and thin filaments. These two sets of planes give rise to the two strongest X-ray reflections, the 1,0 and 1,1 reflections. If mass, in the form of crossbridges, leaves the vicinity of the thick filaments and moves to the vicinity of the thin filaments, there will be a loss of electron density (mass) on the 1,0 planes and a gain on the 1,1 planes with a corresponding decrease in the 1,0 and an increase in the 1,1 reflection intensity. The ratio of the 1,1 equatorial reflection intensity to that of the 1,0 (I_1,1_/I_1,0_), therefore, has been used for a long time to estimate mass transfer of heads from the region of the thick filament to that of the thin filament [32]. I_1,1_/I_1,0_ has been shown to be, in some cases, a linear function of force in contracting mammalian skinned skeletal muscle [34] as well as in intact cardiac muscle [35], where the intensity ratio was shown to correlate well with tension, especially during the early phase of contraction [35]. For these reasons, it is tempting to assume that I_1,1_/I_1,0_ is a direct measure of the number of actin-bound, force-producing crossbridges.

There are serious difficulties with this simple picture, however. Factors that affect the equatorial X-ray reflection intensities have been investigated using crude cylinder models of the sarcomere [8,60,61,62,63]. These include, in addition to mass shifts, lattice spacing, sarcomere length (i.e., degree of thick and thin filament overlap), and degree of ordering of the thick and thin filaments around their lattice positions. For instance, if the thin filament is much more mobile (i.e., less well localized at its lattice position) than the thick filament, this can have a profound effect on I_1,1_/I_1,0_, and this appears to be the case in resting frog muscle [60] at low temperature, and in rabbit muscle at high temperature [63]. Ait Mou et al. 2016 [12] also showed that the thin filaments become better localized when the muscles are stretched, and generate significant passive tension. Furthermore, close vicinity does not mean the myosin heads are actually stereo-specifically attached to actin and generating force [17,18,64].

Estimates of the fraction of the force-generating crossbridges vary widely between species and the methods used to estimate this value. In isometrically contracting frog muscle, for instance, the equatorial intensity ratio, I_1,1_/I_1,0_ can be similar to that of rigor [17,32,33]. The myosin-based layer lines appear to completely disappear, being replaced by actin- and actomyosin-based layer lines [55]. This was interpreted to indicate that up to 80% of myosin heads are in the vicinity of thin filaments in isometric contraction [17]. Studies of the actin-based layer lines, however, suggested that less than 30% of these myosin heads that are close to actin in contraction were stereo-specifically bound in a rigor-like configuration [17,64]. The discrepancy between the equatorial intensity ratio and other estimates of the actual number of crossbridges in frog muscle make estimates of the numbers of force-producing crossbridges problematic in this system.

In mouse skeletal muscle, however, we have demonstrated a linear relationship between the changes in I_1,1_/I_1,0_ changes and maximum isometric tension (Figure 4A). The change in I_1,1_/I_1,0_ from resting was 0.76 in contracting muscle and 1.72 in rigor patterns (calculated from Figure 3C). Assuming a linear relationship of I_1,1_/I_1,0_ to the number of recruited myosin heads, as we see in Figure 4A for maximum isometric tension, this would imply that 55% of the relaxed myosin heads remain in the resting configuration during tetanic contraction, which is very close to the number estimated from the residual MLL4 intensity data (53%). The correspondence between these two numbers, and the near-linearity of the relationships, indicate that either I_1,1_/I_1,0_ or residual MLL4 can be used to estimate the fraction of crossbridges in the resting configuration and, in light of the rigor data, that the remainder of the crossbridges can be reasonably assumed to be associated with actin, and available to generate force. Furthermore, the fraction of this population that is stereo-specifically bound and actually generating force must not be changing appreciably with force level, for the actin associated fraction to have a near-linear relation with force.

There are some caveats with interpreting I_1,1_/I_1,0_ in addition to those discussed above. Bershitsky et al. [64] reported that the thin filaments become better localized at their lattice positions in rigor, which could explain part of the large increase in I_1,1_/I_1,0_ in rigor. One should be cautious, therefore, in assuming that the observed increase of I_1,1_/I_1,0_ over isometric contraction is entirely due to a 3.7-fold increase in the number of attached myosin heads. Bershitsky et al. [63] also made it clear that temperature changes, either steady state or in temperature jump experiments, have a profound effect on the equatorial intensities. In our experiments, the experimental temperatures do not change, and mammals maintain a constant body temperature in vivo, but this could be relevant in some experiments. We have also seen that the degree of disorder of the second kind (σ_s_) is approximately twice as large under contracting conditions than in resting. There is currently no scholarly consensus on how to correct the equatorial intensity ratio (I_1,1_/I_1,0_) for this effect, if indeed it needs to be corrected. The vast majority of I_1,1_/I_1,0_ measurements reported in the literature have had no corrections of any kind applied, as was the case here. The reported width parameters should suffice for any such corrections in the future, should they be deemed to be desirable.

The better statistics in the time-resolved I_1,1_/I_1,0_ as a function of force during rise of tension measurements shown in Figure 5E show, however, that the relationship between I_1,1_/I_1,0_ and force deviates from linearity at high forces, but can still be assumed linear up to 60% of maximum tension (Figure 5F). While detailed modeling will ultimately require understanding the sources of this non-linearity, one can still do useful things with these relationships. For instance, Kiss and his colleagues reported [44] that in a nebulin cKO mouse model that suffers significant muscle weakness, the MLL4 residue in the contracted pattern was higher in nebulin cKO muscle than control muscle, indicating there were fewer crossbridges attached to actin. In any case, the residual MLL4 intensity can be used to parameterize the amount of mass associated with the thick filament to be compared with the estimates of mass associated with the thin filament from I_1,1_/I_1,0_ for a given set of conditions or for a given transgenic model system.

Going forward, it would be highly desirable to determine what fraction of actin-associated myosin heads are truly stereo-specifically bound to actin and force producing. Theoretical and experimental studies by Tsaturyan, Ferenczi, and their colleagues [18,43,57,65] have indicated that this could be done by studying the intensity changes in the first actin layer line. These experiments could be done on mouse muscle with a long X-ray camera, and the results could help resolve remaining ambiguities. For instance, Tsaturyan [18] have estimated the percentage of force producing crossbridges for skinned rabbit psoas muscle to be ~40%. Linari et al. [19] have an even lower estimate, ~30%, based on stiffness measurements. It would be helpful to establish, in the same muscle system, whether the difference between these numbers and the ~45% (100–55% associated with the thick filament) number we get is a species/preparation difference (intact mouse vs skinned rabbit) or that the difference is an indication of the fraction of myosin heads that are closely associated with actin, but are not force producing.

### 3.3. Is There More Than One Quasi-Helically Ordered State in Resting Mouse Muscle?

Under resting conditions, the myosin heads have been proposed to be in either a quasi-helically ordered “super-relaxed” (SRX) or a disordered relaxed state (DRX state) [24]. The resting myosin layer lines can only come from a quasi-helically ordered array of relaxed myosin heads, and if SRX is the only ordered state, this would mean that the MLLs only arise from heads in the SRX state. We would expect, therefore, no change in the position of the maxima on the MLL4 spacing and, hence, radius to the center of mass of the crossbridges (R_m_) with blebbistatin treatment. Our results showed, however, that R_m_ was smaller with the treatment of blebbistatin than in the normal resting state (Figure 6A). In the presence of blebbistatin, therefore, myosin heads are being held closer to thick filament backbone, consistent with the observed decrease in I_1,1_/I_1,0_ in relaxed muscle (Figure 5B).

The change in R_m_ upon treatment with blebbistatin is 0.2 nm, and might seem insignificant. The diameter of the thick filament backbone is estimated to be about 9–10 nm [66]. Due to the steric hindrance of the thick filament backbone, the maximum excursion of the myosin heads with respect to the thick filament backbone would be ~2.5–3.5 nm. Given the data in Figure 6B, the smallest R_m_ was about 11 nm in highly stretched muscle, so a 0.2 nm movement accounts for at least 6–8%, and more likely, 13% of total range of movement relative to the thick filament backbone, which is not negligible. Given the existence of a helically ordered state with a larger R_m_, this indicates that either the DRX is not really disordered, or there is at least one other relaxed state, perhaps a “regular relaxed state” (RRX), that is sufficiently ordered to give rise to MLLs. In support of this notion, the average myosin head radii (Figure 6A), as well as the layer line intensities, (Figure 4A) were the same with or without treatment by BTS. It would seem, therefore, that BTS inhibits muscle contraction without shifting myosin heads to a super-relaxed state, or at least, not to the same highly ordered state stabilized by blebbistatin.

### 3.4. Interconversion between the Blebbistatin-Stabilized SRX State and Other States

In mouse muscle treated with blebbistatin, we see a modest decrease in I_1,1_/I_1,0_ (Figure 5B), a modest decrease in the myosin head radius, R_m_ (Figure 6A), and a two-fold increase in the intensity of MLL4 (Figure 4A). The decrease of R_m_ and an increase in I_10_, (leading to a decreased I_1,1_/I_1,0_) with blebbistatin treatment is likely to be due to the tighter packing of the heads on the surface of the thick filament and a compaction of the structure of the heads when they adopt the IHM. A given X-ray reflection can increase in intensity if there is more mass associated with the structures giving rise to it, e.g., by increasing the number of molecules adopting a particular configuration. Alternatively, it could because an unchanged number of diffracting objects become better ordered. The observed increase in the intensity of MLL4, therefore, could either be from the recruitment of heads from the DRX state to the SRX state, where the heads adopt the IHM, or it could be from an existing population of helically arranged heads simply becoming more ordered. Given that R_m_ is different in the presence and absence of blebbistatin, this is consistent with two populations of quasi-helically ordered crossbridges that can interconvert, perhaps by the formation or disruption of the IHM. If so, the regularity of the head arrangement in IHM could lead to most, if not all, of the observed increase in the intensity of MLL4 with blebbistatin treatment. The presence of multiple populations of heads at any given incubation time is likely to be the cause of the non-linearity of the relationship between MLL4 intensity and active tension varied by incubating intact EDL muscle with blebbistatin (Figure 4B) for various lengths of time. In BTS-treated muscle, inhibition does not appear to involve large-scale structural changes, and a linear relationship (Figure 4A) of layer line intensity and force may be expected since all that is changing is the number of heads able to bind to actin.

Our findings in membrane-intact, living mouse muscle treated with blebbistatin are broadly similar to those reported recently by Iwamoto [48] in his study of the effect of myosin inhibitors on skinned rabbit psoas muscle. The main observations with treatment with blebbistatin were a substantial intensification of myosin layer lines, and a reduction of I_1,1_/I_1,0_ under relaxing conditions. At pCa4, he observed an increase in I_1,1_/I_1,0_, a substantial reduction in MLL intensity and a substantial increase in the outer portion of the second actin layer line (ALL2) at pCa4. The latter reflection greatly intensifies during contraction in striated muscle, and is attributed to tropomyosin moving closer into the groove of the actin helix uncovering the myosin binding sites on actin, allowing heads to bind [53,67,68,69,70,71]. This observation was early evidence for the so-called steric blocking model of muscle regulation [71,72]. On this basis, the author concluded that activation processes occur normally in blebbistatin-treated muscle, but force is reduced because of the heads being trapped in a pre-powerstroke state, as suggested by biochemical studies. Blebbistatin appears to bind in a hydrophobic pocket at the apex of the 50 kDa cleft of the myosin motor domain [73], and this binding has been proposed to stabilize the structure in a “transition” state, preceding the force-producing step after actin binding [74].

A difficulty with this interpretation is that in Iwamoto [48], the increase in I_1,1_/I_1,0_ in blebbistatin-treated muscle under contracting conditions is modest, just enough to bring it close to the relaxed value without blebbistatin treatment, so it is not clear that this indicates that substantial populations of actin-associated myosin heads are being formed in the presence of calcium. In our data from intact mouse muscle, there is no change in I_1,1_/I_1,0_ upon electrical activation of fully inhibited muscle by either blebbistatin (Figure 5C) or BTS (Figure 5D). Furthermore, we also do not see substantial weakening of myosin layer lines under contracting conditions (Figure 4B). We also see only a modest intensification of Tm/ALL2 upon activation, much less than in the absence of inhibitor (Figure 7). There was no difference in integrated Tm reflection intensities between normal contracting soleus and EDL muscle, but the Tm integrated intensities were much smaller in fully inhibited muscle than in normal contracting muscle. This is consistent with the notion that in intact mouse skeletal muscle, at near physiological temperatures, strong binding of myosin to actin is needed to move tropomyosin into the fully open state [71,72].

It needs to be noted that Iwamoto’s study was done at low temperature (6–8 °C) in skinned muscle, and the current study on intact muscle was done at 22 °C, where the ordering of the crossbridges in either the resting or the relaxed state is known to be much better. It is also closer to physiological temperature of 37 °C. It seems then, that at high temperatures, blebbistatin-treated myosin heads appear to be predominantly in a highly ordered and stable quasi-helical arrangement of IHMs around the thick filament backbone that are unable to make contact with the thin filament. This appears not to be the case at low temperatures, as used in Iwamoto’s study. Both of the aforementioned biochemical studies were done on isolated myosin. Rahman et al. recently reported mechanical studies in skinned rabbit psoas muscle fibers at 5 °C, where they were able to perform isometric force and respond to ramp stretch experiments, indicating that the heads were able to interact with actin and were not closely associated with the thick filament backbone at this low temperature [75]. Another potential source of variability in all these studies is the unknown phosphorylation levels of myofilament proteins. For example, Stewart et al. [76] showed that in the presence of blebbistatin, maximal shortening velocities at 30 °C, were decreased in fibers with phosphorylated regulatory light chains on myosin, but were not inhibited in dephosphorylated fibers. For another example, Colson et al. [77] showed that phosphorylation of cMyBP-C increased I_1,1_/I_1,0_, presumably by relieving a constraint on the crossbridges, thereby increasing the proximity of myosin to binding sites on actin.

Xu et al. [47] also reported intensification of the myosin layer lines in skinned rabbit psoas muscle stretched beyond filament overlap, but did not see a shift in the MLL1 first maximum indicative of a change in Rm with blebbistatin treatment. It is not clear whether this is due to a genuine species difference, the fact that this study was done on skinned, overstretched muscles, or if a change in Rm would have been detected by examining more, higher quality images. A possible explanation for this lack of change in Rm with blebbistatin treatment is suggested by our passive stretch experiments that indicate that at long sarcomere lengths, the heads are already close to the backbone (Figure 6B) such that blebbistatin treatment would have little additional effect.

The mechanisms behind the observed reduction in Rm with stretch are not completely clear. Many vertebrate-striated muscles display constant lattice volume behavior, except under extreme conditions [78], so this is likely to be the case here. Verification of constant lattice volume is not possible from the current experimental data because of the difficulty of laser diffraction on whole intact muscle, but could be addressed in future experiments. Constant volume behavior of the lattice would predict a reduction in lattice spacing at long sarcomere length as observe in experiments on intact cardiac muscle [12,79]. Ait Mou et al. [12] reported that under diastolic conditions in twitching intact rat trabeculae, both the intensity ratio and Rm indicated that the majority of the heads are held close to the thick filament backbone, an unexpected finding in light of the enhanced contractility at long sarcomere lengths due to myofilament length-dependent activation, but consistent with our findings. Passive tension was observed by Fusi et al. [80] to reconfigure approximately 15% of the heads, presumably due to increasing thick filament strain, from an orientation approximately parallel to the filament axis to one perpendicular to filament axis. This was at a passive tension of ~30% of max isometric tension, similar to the highest passive tensions seen in our study. This would imply that 85% of the heads remain close to the backbone, and 15% of the heads perpendicular to it. When averaged in the X-ray diffraction patterns, this would be expected to result in a modest smearing on the layer line intensities towards lower radial spacings. This would be hard to detect given the relatively low precision of the layer line measurements. Together, these results would imply that at the small lattice spacings and high passive tensions at long sarcomere lengths, the majority of the heads are somehow held close to the thick filament backbone, either simply by confinement of the crowded myofilament lattice or perhaps by electrostatic repulsion between the negatively charged myosin heads and actin thin filaments [78].

Evidence is mounting that the SRX state is of clinical significance. It seems that many myosin mutations cause the hypercontractility observed in hypertrophic cardiomyopathy by promoting a shift from an off-state of myosin heads, presumably the SRX, to an on-state now able to bind to actin [81,82]. The small molecule drug candidate mavacamten has been found to decrease contractility and suppress the development of hypertrophy and fibrosis in HCM mouse models [83]. Mavacamten appears to reduce the basal release rates of ADP and Pi [84], by stabilizing the myosin heads in an SRX state [23], similarly to blebbistatin. In support of this notion, we have shown that mavacampten significantly increases the myosin layer line intensities in relaxed skinned porcine cardiac muscle [23], consistent with an enrichment of the quasi-helically ordered SRX state.

Mutations in cardiac myosin binding protein C (cMyBP-C) have been shown to be a major cause of inherited forms of HCM [85,86]. Mice deficient in cardiac myosin binding protein C showed significantly reduced SRX as compared to wild type [87] indicating that competent myosin binding C may be required for regulation of the transition from DRX to SRX in humans. Human cardiac muscle samples from HCM patients with mutations in the cMyBP-C gene (*MYBPC3*) also showed a decreased number of heads in the SRX, as well as decreases in the lifetime of ATP turnover [88]. Dephosphorylation of cMyBP-C has been shown to promote or stabilize the relaxed/super-relaxed quasi-helical ordering of the myosin heads on the filament surface, whereas phosphorylation weakens this stabilization and binding of the heads to the backbone [89], indicating that this is an important part of the regulatory mechanism. Due to its sensitivity to shifts in the populations of ordered heads, it is likely that the residual MLL4 intensity assay in mouse skeletal muscle could be a useful method to investigate some aspects of these processes in future studies.

## 4. Materials and Methods

### 4.1. Muscle Preparations

All animal experiments were governed by protocols approved by the Illinois Institute of Technology Institutional Animal Care and Use Committee (Protocol 2015-001, Approval date: 3 November 2015) and followed the “Guide for the Care and Use of Laboratory Animals” [90]. The mice used in these experiments were C57BL/6 mice (male, 10–15 weeks). Mice were euthanized by carbon dioxide inhalation and cervical dislocation. The skin was removed from the hind limbs and the muscles were exposed. Muscles were kept moist by applying Ringer’s solution (145 mM NaCl, 2.5 mM KCl, 1.0 mM MgSO_4_, 1.0 mM CaCl_2_, 10.0 mM HEPES, 11 mM glucose, pH 7.4) to the muscle at regular intervals. The limbs were separated and pinned down in a petri dish with Ringer’s solution perfused with 100% oxygen. The soleus and EDL muscle were dissected out. One end of the muscle was hooked to a combined servo motor/force transducer (300C_LR, Aurora Scientific Inc., Aurora, ON, Canada), mounted on an X–Y–Z positioner, and the other end was hooked in the muscle chamber. Muscles were bathed in Ringer’s solution and continuously bubbled with oxygen throughout experiments. Experiments were done at room temperature (22 °C).

For rigor preparations, the muscles were dissected as described above. They were then pinned down and pre-stretched to *Lo*, by dissecting pins to an agar surface to prevent shortening during rigor force development. They were then incubated in Ringer’s solution with 1% iodoacetic acid (Sigma, St Louis, MO, USA) for 24 h [91]. The muscle was mounted in the chamber and stretched by about 10%, as suggested by Huxley [91], before taking X-ray patterns.

### 4.2. X-ray Diffraction

X-ray diffraction experiments used the BioCAT beamline 18ID at the Advanced Photon Source, Argonne National Laboratory [92]. The X-ray beam energy was set to 12 keV (0.1033 nm wavelength) at an incident flux of ~10^13^ photons per second in the full beam. The X-ray beam was focused to ~130 × 60 µm at the detector. Except for patterns shown in Figure 2, the specimen to detector distance was about 2 m. For the data shown in Figure 2 it was 9 m. The experiment was controlled by an ASI 610A data acquisition and control system (Aurora Scientific Inc., Aurora, ON, Canada). The muscles were activated for 1.5 s (100Hz pulse frequency, 2 ms pulse width) tetanus followed by a 1 s resting period during which data was collected continuously at 500 Hz. There was at least a 10 min waiting period between subsequent tetani to allow the muscle to recover. To minimize the radiation-induced muscle damage, the experimental cell was oscillated along its vertical and horizontal axes, independently, with a translational velocity of 10 mm/s. The velocity matched the X-ray beam dimensions so that overlapping X-ray exposure on the muscle surface could be avoided during data collection. To avoid rundown of the preparation only, data from the first contraction in the X-ray beam was used for time-resolved experiments. In the inhibitor experiments using blebbistatin or BTS, no more than two contractions were used. The beam position on the sample was translated between contractions to avoid irradiating the same spot. The X-ray shutter was kept open during the entire protocol with the detector (Pilatus 3 1M, Dectris Inc., Baden, Switzerland), continuously collecting data frames with a 1 ms exposure time and 1 ms readout time per frame. For experiments with inhibitors, resting and contracting X-ray patterns were collected first in Ringer’s solution. Inhibitor, either 75 µM blebbistatin or 100 µM BTS, was then added into ringer solution, and X-ray patterns were then collected every 10 min until the force dropped to less than 5% of maximum isometric tension in the untreated muscle. In passive stretch experiments, the muscle was set to L_0_, and the muscle was stretched, at a constant speed (10%/min), to 15%, 25%, and 35% over L_0_. One-hundred X-ray images each, with a 10 ms exposure time and 100 ms readout time were taken at each of the four different lengths.

### 4.3. Post-Experiment Muscle Treatment

After each mechanical and X-ray experiment, the muscle was recovered for cross-sectional area calculation. The cross-section area was calculated according to Alexander et al. [93] using measured muscle lengths and the muscle masses assuming a muscle density of 1.06 g/ml [94,95]. The muscle was placed back in relaxation solution, and was then stretched to the experimental length and fixed in 10% formalin for 10 min. The sarcomere length was recorded by using a video sarcomere length system (model 900B, Aurora Scientific Inc., Aurora, ON, Canada) from fixed fiber bundles throughout the entire muscle cross-section [94].

### 4.4. Data Analysis

X-ray images collected during 1 s of resting and 1 s of isometric tetanus plateau were summed, respectively, using FIT2D [96]. The data were analyzed using data analysis programs belonging to the MuscleX open-source software package developed at BioCAT [97]. The images were quadrant folded and background subtracted assuming a circularly symmetric model for the diffuse background using the “Quadrant fold” routine in MuscleX.

The interfilament lattice spacings and intensity ratios were measured by the “Equator” routine in MuscleX. The equatorial intensity trace was reduced to a one-dimensional projection by drawing a box along the equator with a width set to be just outside the reflections. The first 5 diffraction orders were modeled on Gaussian functions superimposed on a smooth background. The diffuse background was removed using a convex hull organization, and the intensity distribution along the equator was fit using a Marquardt–Levenberg algorithm assuming a series of Gaussian functions. The width of a given peak, σ_h,k_, may be expressed as $\sqrt{\left(\sigma_{c}{}^{2}+\sigma_{d}{}^{2}\theta_{\mathrm{hk}}{}^{2}+\sigma_{s}{}^{2}\theta_{k}{}^{4}\right)}$, where $\theta_{\mathrm{hk}}=\sqrt{\left(h^{2}+k^{2}+\mathrm{hk}\right)}$ [49,61], σ_c_ is the known width of the X-ray beam (set to 1 pixel on the detector which is equivalent to 0.84 × 10^−3^ nm^−1^ under our conditions), σ_d_ is related to amount of heterogeneity in interfilament spacing (Δd_10_/d_10_) among the myofibrils, and σ_s_ is related to the amount of paracrystalline (liquid-like) disorder of the myofilaments in the hexagonal lattice [49]. Liquid-like disorder was also called “disorder of the second kind” [98]. σ_s_ can be expressed in terms of ΔX/d_10_, where ΔX is the standard deviation in the distribution of distances between nearest-neighbor unit cells in a given myofibril [49]. σ_s_ increased substantially during contraction in skeletal muscle [49].

The spacing between first maxima (used to calculate R_m_ [12]) and intensity of MLL4 were measured by the “Projection Traces” routine in MuscleX. Using this program, the intensity was integrated along a layer line and projected onto a line perpendicular to the meridian. The box width for the MLL4 intensity integration was chosen to be from 0.088 to 0.097 nm^−1^, parallel to the equator, as shown in Supplemental Figure S1. This integration region was chosen to minimize the contribution of AM_+1_ to the integrated intensity. An example of the resulting intensity trace is shown in Supplementary Figure S2. The background is modeled as the sum of three Gaussian peaks, one broad and diffuse for the overall background, a narrower Gaussian for the background just around the meridian, and an additional Gaussian at the center for the meridional peak itself. The layer line intensity around the first maximum was modeled as a single Gaussian peak on top of this background. These functions were fit simultaneously to yield the centroid of the first maximum peak on the layer line and the total intensity of the peak.

In order to compare the intensity changes of diffraction features taken under different conditions, the diffraction images were normalized by dividing the measured diffraction intensities by the sum of the intensities in the diffuse background images calculated by the Quadrant Fold program.

It should be noted that there are other factors that could affect the MLL4 and equatorial intensities. When activated under fixed end conditions, muscles will initially shorten by some small amount. This would increase the amount of diffracting mass in the beam (by increasing the number of sarcomeres) which increase affect the intensities. The degree of shortening in frog muscle single fibers under fixed end conditions can be seen in the data of Burton et al. [99] and are of the order 4–6%. Assuming the initial shortening in our preparations were similar, this would have only a small effect. Since we could not measure sarcomere length during contraction, we could not monitor the degree of shortening in our experiments, so no correction for this effect was attempted.

### 4.5. Statistics

Statistical analyses were performed using GraphPad Prism 7 (GraphPad Software, La Jolla, CA, USA). The results are showed here are as mean ± SEM unless otherwise stated. Symbols on figures: ns: *p* ≥ 0.05, *: *p* < 0.05, **: *p* < 0.01, ***: *p* < 0.001 and ****: *p* < 0.0001 for two-tailed Student’s *t*-tests.

## 5. Conclusions

We have shown that mouse skeletal muscle can produce high quality X-ray diffraction patterns containing broadly similar diffraction features as those from much better studied systems, such as frog leg muscles or skinned rabbit psoas muscle. Given the availability of transgenic mouse models for a wide range of inherited muscle diseases, these findings suggest that the intact mouse skeletal muscle preparation from normal and transgenic animals can provide a powerful experimental platform for testing hypotheses concerning the structural basis of the relationship of mutations in sarcomeric proteins to disease phenotype. The presence of residual myosin layer line intensities in contracting mouse muscle patterns provides an additional tool, along with the I_1,1_/I_1,0_ intensity ratio, for estimating the proportions of active versus relaxed myosin heads under a given set of conditions that can be used to characterize a given physiological condition or mutant muscle type. In particular, changes in MLL4 intensity, and in the myosin head radius, R_m_, may be used to study the role of the SRX state in myosin regulation.

## Figures and Tables

**Table 1:** Peak width parameters for the equatorial reflections in resting and normal contracting muscle.

Equatorial Width Parameters	Resting (*n* = 9)	Contraction (*n* = 9)	*p* Value
Axial width (nm^−1^)	2.7 ± 0.2	2.8 ± 0.2	ns
σ_d_ (nm^−1^)	1.36 ± 0.09 (×10^−3^)	2.21 ± 0.11 (×10^−3^)	****
σ_s_ (nm^−1^)	0.54 ± 0.04 (×10^−3^)	0.98 ± 0.08 (×10^−3^)	**

ns: *p* ≥ 0.05; ****: *p* < 0.0001; **: *p* < 0.01.

## Supplementary Materials

Supplementary materials can be found at http://www.mdpi.com/1422-0067/19/9/2643/s1.

## Abbreviations

ALL	actin layer line
AM	actomyosin
BB	blebbistatin
BTS	*N*-benzyl-p-toluene sulfonamide
C1	lowest angle myosin binding protein C reflection
C2	second myosin binding protein C reflection (doublet with C1)
C4	4th myosin binding protein C reflection (doublet with C3)
cKO	conditional knockout
DRX	disordered-relaxed state
EDL	extensor digitorum longus muscle
MLL	myosin layer line
M1	first order myosin meridional X-ray reflection
M2	second order myosin meridional X-ray reflection
M3	third order myosin meridional X-ray reflection
M6	sixth order myosin meridional reflection
RRX	regular-relaxed state
Tm	tropomyosin
Tn1	first order troponin meridional reflection
Tn2	second order troponin meridional reflection
Tn3	third order troponin meridional reflection
