# Supplementary material for: Myosin Head Configurations in Resting and Contracting Murine Skeletal Muscle
Source: Int J Mol Sci. 2018 Sep 6;19(9):2643. doi: 10.3390/ijms19092643 (PMC6165214; doi:10.3390/ijms19092643)
Supplement: Supplementary file 1 [file ijms-19-02643-s001.pdf]

Supplementary materials

Myosin Head Configurations in Resting and Contracting Murine Skeletal Muscle

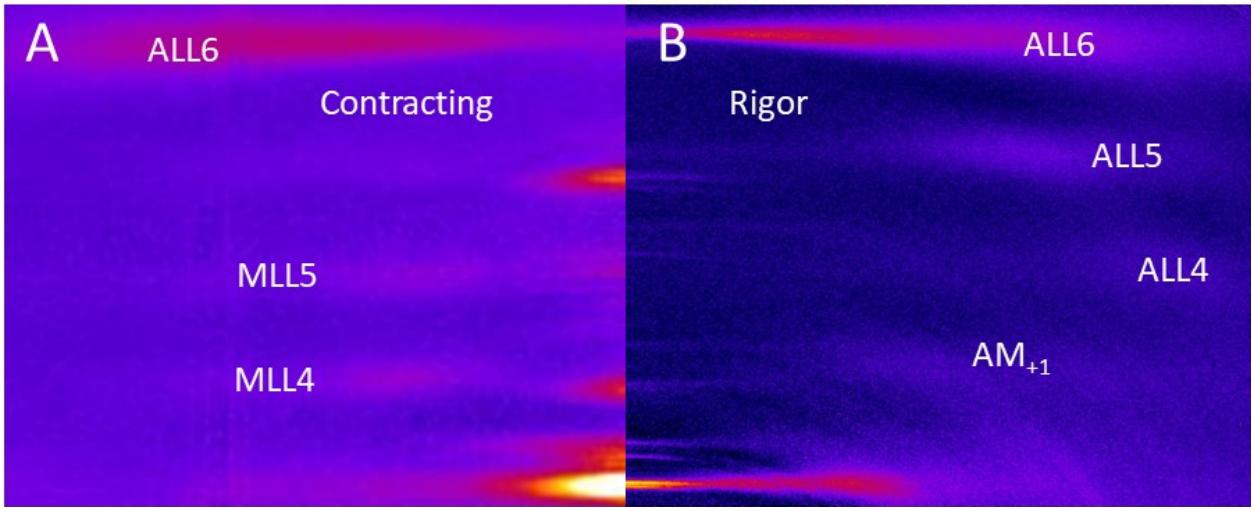

Figure S1. Myosin layer lines in a contracting pattern from mouse EDL muscle and Actin layer lines in a rigor muscle pattern. (**A**) The MLLs were visible in contracting pattern but ALL and AM layer lines were very weak. (**B**) MLLs have disappeared in the rigor pattern while ALLs and AM layer lines were present at higher axial and radial spacing.

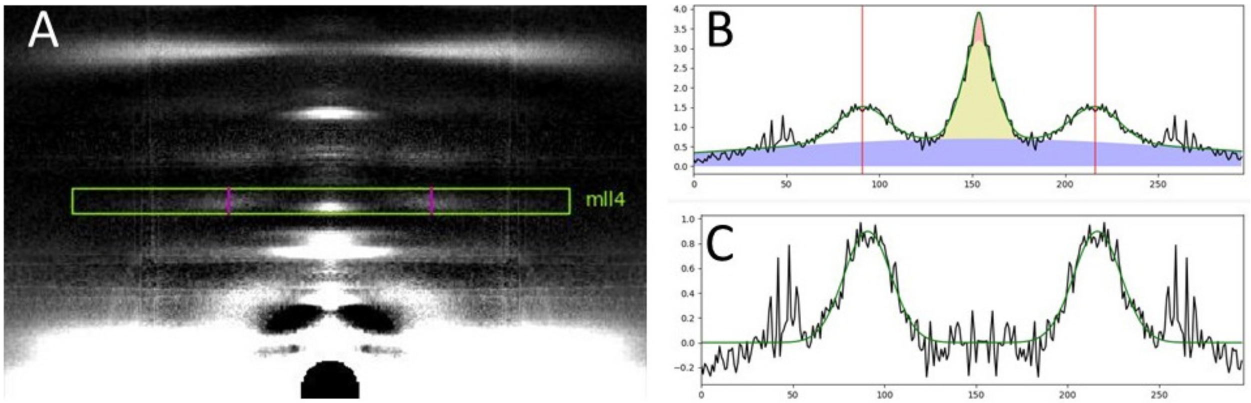

Figure S2. MLL4 integration area and one dimensional intensity profile. The green box in panel (**A**) was the integration area selected for MLL4 and the vertical lines (vertical lines inside green box in Panel A and vertical lines in Panel B) indicate the centroids on either side of MLL4. Full (**B**) and background subtracted (**C**) MLL4 intensity profile. The blue and beige gaussian peaks are background. The small orange peak is the meridional reflection. The MLL4 intensity profile in **C** was fitted with Gaussian functions (shown as green line in Panel B and C). The noise to the left and right of the layer line peaks are detector artefacts.
